# Heterogeneous Electrochemical Immunoassay of Hippuric Acid on the Electrodeposited Organic Films

**DOI:** 10.3390/s141018886

**Published:** 2014-10-13

**Authors:** Young-Bong Choi, Nam-Hyuk Kim, Seung-Hoi Kim, Gun-Sik Tae, Hyug-Han Kim

**Affiliations:** 1 Department of chemistry, College of Natural Science, Dankook University, Anseo-Dong, Cheonan 330-714, Korea; E-Mails: chem0404@dankook.ac.kr (Y.-B.C.); albireo0827@naver.com (N.-H.K.); kimsemail@dankook.ac.kr (S.-H.K.); 2 Department of biology, College of Natural Science, Dankook University, Anseo-Dong, Cheonan 330-714, Korea; E-Mail: gtae@dankook.ac.kr

**Keywords:** hippuric acid, toluene, electrochemical immunosensor

## Abstract

By directly coordinating hippuric acid (HA) to the ferrate (Fe) as an electron transfer mediator, we synthesized a Fe-HA complex, which shows a good electrochemical signal and thus enables the electrochemical immunoanalysis for HA. We electrodeposited organic films containing imidazole groups on the electrode surface and then bonded Ni ion (positive charge) to induce immobilization of Fe-HA (negative charge) through the electrostatic interaction. The heterogeneous competitive immunoassay system relies on the interaction between immobilized Fe-HA antigen conjugate and free HA antigen to its antibody (anti-HA). The electric signal becomes weaker due to the hindered electron transfer reaction when a large-sized HA antibody is bound onto the Fe-HA. However, in the presence of HA, the electric signal increases because free HA competitively reacts with the HA antibody prior to actual reaction and thus prevents the HA antibody from interacting with Fe-HA at the electrode surface. This competition reaction enabled an electrochemical quantitative analysis of HA concentration with a detection limit of 0.5 μg mL^−1^, and thus allowed us to develop a simple and rapid electrochemical immunosensor.

## 1. Introduction

Toluene is a broadly applied compound in chemical synthesis, in paints, in detergents, in adhesives, and in the petroleum industry. Those who are exposed to toluene for a long time have been found to suffer from anatomical changes in the brain. Simple monitoring of exposure to toluene is very important in occupational health care. Hippuric acid (HA), which is a major component of toluene metabolites, is a chemical compound with a molecular weight of 178.17 Da. Urinary HA concentration is widely used as a “target compound of toluene exposure” [1–3] and is measured by radioimmunoassay, enzyme linked immunosorbent assay (ELISA), UV-Visible spectroscopy, gas chromatography, and high performance liquid chromatography (HPLC). Among of these methods, HPLC-based analysis currently represents the most popular technique due to its simplicity and the fact that it does not involve radioactivity [4–7]. However, it is unsuitable for simultaneously analyzing a large number of samples and requires expensive equipment and trained technicians. To allow the measurement of many samples simultaneously, the ELISA technique was developed and has been used. However, it involves the use of expensive chemicals, such as enzymes and staining reagents, and equipment, such as an ELISA washer and an ELISA reader, as well as highly skilled lab personnel. The electrochemical immunosensor is a useful tool for determining toluene exposure in industrial and environmental settings or substance abuse, such as glue-sniffing, that overcomes these disadvantages and allows analysis of chemical compounds qualitatively and quantitatively without the need for specialized skills [8–11].

Electrochemical immunoassays have many advantages over conventional immunoassays, such as simple instrumentation, relatively low cost, miniaturization, portability, disposability, and full automation, and have therefore attracted keen interest from researchers [12–15]. Particularly interesting are electrochemical immunoassays that use metals or metal nanoparticles and have the advantages of high sensitivity and rapid electron transport reaction due to the increased electrode surface area and conductivity. Among different metals used for electrodes, Ni is less expensive than Pt or Au and is easier to obtain. In addition, it has superior electrical oxidation abilities when reacting with oxides. Also, Ni ions can easily bind to an imidazole group and are broadly used in immobilization of substances containing the latter [16–19].

The electrode plays a pivotal role in the electrochemical immunosensor. There are two methods of measurement depending on the use of the electrode: homogenous and heterogeneous. In the easy-to-implement and inexpensive homogeneous method, the antigen-antibody reaction takes place in a solution in which the measurement is consequently performed. The major drawback to this method is its low sensitivity arising from the difficulty of determining whether the reaction between antigens and antibodies actually took place [20–23]. The heterogeneous method requires a more complicated procedure because antigens or antibodies must be first immobilized on the electrode. However, this disadvantage is outweighed by the advantages, such as increased surface area and improved conductivity resulting in enhanced signal integrity. This method leads to improved sensitivity and is widely used in immunological reactions [24,25]. Electrochemical immunoassays are often used in the measurement of blood or urine samples that contain interfering substances such as ascorbic acid, uric acid, ammonia, and urea [26]. It is usually very difficult to distinguish the signal of the target substance from the signals of the interfering substances if the latter are detected during the measurement. In order to produce a highly selective electrochemical immunosensor, electrode surface treatment is necessary to prevent the reaction of the interfering substances with the electrode. One of the common methods is to block the electron transfer signals of the interfering substances using organic films. The problem with this method is that the application of the organic films to the electrode leads to a decrease of the signal of the target substance. Therefore, selective signal transmission methods using metal or metal nanoparticles are generally preferred [27,28].

**Figure 1.**
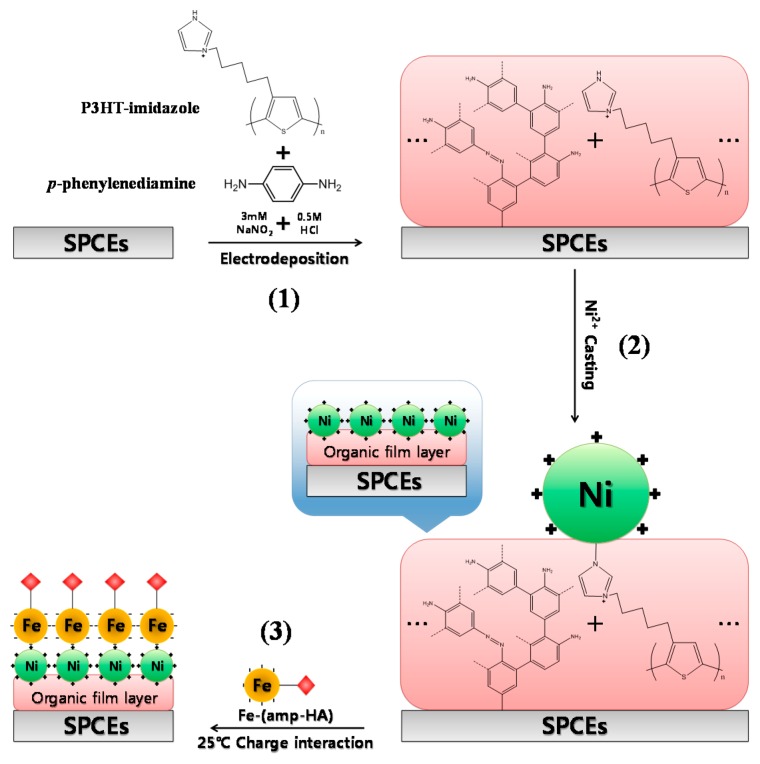
Schematic representation of the fabricating SPCEs/Organic film/Ni/Fe-HA for heterogeneous electrochemical immunoassay.

Compared to our previous papers, the benefits of the present paper include (1) the easy immobilization of Ni ions onto the electrode; (2) the prompt synthesis of ferrate antigen; (3) less interference due to the electrostatic interaction between Ni(II) and ferrate; and (4) a relatively low cost [29–31]. In this study, we used electrochemical techniques to form organic films containing imidazole rings on the electrodes (Figure 1(1)). Taking advantage of the high affinity between imidazole rings and Ni ions, we succeeded in electrodepositing Ni ions (+ charge) on the organic films (Figure 1(2)). Similar to other metal catalysts, Ni ions increased the surface area exhibiting superior electron transfer performance and thus canceling out the signal reduction induced by the inhibition of electron transfer by the organic films. In order to immobilize antigens on Ni ions (+ charge), we synthesized a Fe-HA complex containing Fe as an electron transfer mediator and HA as an antigen. As shown in Figure 1(3), antigens were finally immobilized onto the electrodes through the charge interaction between Ni ions (+ charge) and Fe-HA (− charge), thus minimizing the influence of other interfering substances because only the signal from the HA-tagged Fe complex could be measured. The reaction with anti-HA (HA antibody) was maintained through the Fe-HA-immobilized electrode. Once anti-HA was bound to the electrode through the antigen-antibody reaction, signal intensity was reduced due to the Fe-HA's electron transfer inhibition. Based on this phenomenon, we fixed the anti-HA concentration, varied the HA concentration and observed the competition reaction between HA and Fe-HA immobilized on the electrode surface. As a result, we confirmed that an increase in HA concentration led to a signal increase because anti-HA could no longer bind to the electrode surface. This discovery led to the development of the electrochemical immunosensor capable of quantitatively analyzing HA.

## 2. Experimental Section

### 2.1. Reagents

A carbon electrode was screen-printed on an overhead projector (OHP) film (Electrodag 423SS, Acheson, Port Huron, MI, USA) using a screen printing machine (BS-860AP, Bando, Seoul, Korea). Monoclonal anti-HA (HA antibody) was kindly donated by HBI (Seoul, Korea). 4-aminomethylpyridine, HA, *p*-phenylenediamine, sodium nitrite, nickel chloride(Ⅱ) hexahydrate, buffering salts, and other chemicals were purchased from Sigma-Aldrich Co. (Milwaukee, WI, USA). Ammonium disodium pentacyanoamminferrate dehydrate was purchased from Fluka. Phosphate-buffered saline (PBS, 4.3 mM NaH_2_PO_4_, 15.1 mM Na_2_HPO_4_, and 140 mM NaCl) and all other solutions were prepared using deionized Milli-Q water (Millipore, Bedford, MA, USA). All chemicals used were of analytical grade.

Poly{3-[6-(1-methylimidazolium-3-yl)alkyl]thiophene-2,5-diyl bromide} (P3HT-imidazole), a conductive polymer, was synthesized following a procedure described in previous papers [32,33].

### 2.2. Preparation of [Fe(CN)_5_(amp-HA)]^3−/2−^ (Fe-HA)

The complex of [Fe(CN)_5_(amp-HA)]^3−/2−^ (Fe-HA) was synthesized as described previously [34]. Fifty milligrams of Na_2_[Fe(CN)_5_NH_3_]·2H_2_O and 44.4 mg of amp-HA were dissolved in 60 mL of aqueous ethanol solution (v/v: 50/50) and kept at room temperature for 24 h. After filtering, the solution was added to 2.0 L of diethyl ether with vigorous stirring. The product was precipitated, washed with ethanol and then dried for several hours in a vacuum oven.

**Figure 2.**
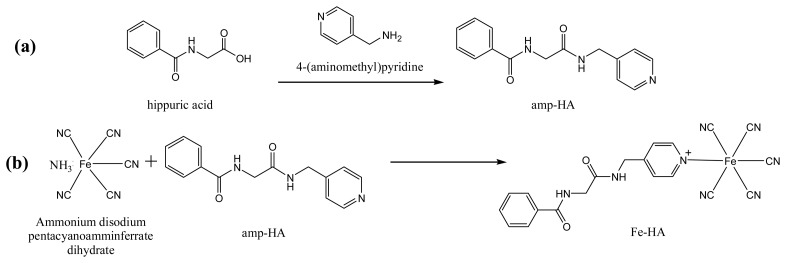
Preparation of Fe-conjugated HA antigen [Fe(CN)_5_(amp-HA)]^3−/2−^ (Fe-HA).

The synthesis of amp-HA (Figure 2a) was confirmed by thin-layer chromatography (TLC) and ^1^H NMR spectroscopy. The ^1^H NMR spectrum (400 MHz, DMSO) was as follows: δ_ppm_ 7.45–7.95 (m, 5H, phenyl), 7.32–8.65 (m, 4H, Py), 3.98–4.25 (t, 4H, *J* = 7.0 Hz, -CH_2_-CH_2_-), 7.8–8.01 (m, 2H, -NH-NH-). The conjugation of Fe-HA (Figure 2b) was verified by UV-vis and FT-IR spectroscopy.

### 2.3. Electrochemical Measurements

Electrochemical measurements were carried out with a CH Instruments model 660A electrochemical workstation (CH Instrument, Austin, TX, USA), interfaced to a computer. The electrochemical characteristics of Fe-HA were studied using 3.0 mm-diameter screen printed carbon electrodes (SPCEs) as the working electrodes. An Ag/AgCl micro-reference electrode (3.0 M KCl, Cypress, Lawrence, KS, USA) scrolled with a 0.5 mm diameter platinum wire counter-electrode was used.

### 2.4. Formation of Organic Films Containing Imidazole Rings on SPCEs

In order to form organic films containing imidazole rings on SPCEs, we prepared a conductive polymer solution by mixing 5 mL of a 3 mM solution of p-Phenylenediamine (3.24 mg) in 0.5M HCl (10 mL) and 5 mL of the conductive polymer P3HT-imidazole (50 mg) in Milli-Q water (10 mL). Sodium nitrate (2.83 mg) was added to the solution and dissolved. After doping 40 μL of the resulting solution onto the SPCEs, 5–30 electrodeposition cycles were performed in order to form the organic films on the surface of the SPCEs. In order to bind Ni ions to the imidazole rings in the organic films, 50 μL of 100 mM NiCl_2_ solution (NiCl_2_·6H_2_O dissolved in Milli-Q water) was doped on the SPCEs formed previously and dried at room temperature for 30 min. The electrodes were then washed with Milli-Q water and dried at room temperature (25 °C). The final step in the electrode production process was the electrodeposition of 40 μL of 5 mM Fe-HA onto the surface of the SPCEs containing immobilized Ni ions, followed by drying at room temperature (25 °C) for 60 min, washing with Milli-Q water, and drying again at room temperature (25 °C). The DPV signal of the SPCEs was tested by measuring the intensity for each scan rate (0.01–0.2 Vs^−1^). The electrolyte used in this measurement was 1 × PBS (pH 7.0) containing 1.0 M NaCl, and the range of the measurement potential was 0.4–2.0 V.

### 2.5. Immune Reaction between Fe-HA Immobilized on the Electrode and Anti-HA

Using the electrostatic interaction method, 40 μL of the synthesized Fe-HA was electrodeposited on the electrode with immobilized Ni ions on it and then washed. The electrode was dried at room temperature and then casted with 40 μL of anti-HA of different concentrations (0–5 mg/mL). The dependence of electrochemical characteristics on the anti-HA concentration was monitored using differential pulse voltammetry (DPV). The results of DPV from 0.1 to 0.8 V *vs*. Ag/AgCl with a pulse amplitude of 0.05 V and a pulse width of 50 ms were collected and plotted as the electrochemical immunosensor signal.

### 2.6. Competition Reaction between Immobilized Fe-HA and Free HA

Twenty microliters HA of different concentrations (0–5 mg/mL) followed by 20.0 μL anti-HA at a concentration of 1.0 mg/mL were casted on the electrode, on which Fe-HA was immobilized. The effect of different HA concentrations on electrochemical characteristics was measured by DPV after an initial delay of 30 s to allow the reaction to take place. The results of DPV from 0.1 to 0.8 V *vs*. Ag/AgCl with pulse amplitude of 0.05 V and a pulse width of 50 ms was collected and plotted as the electrochemical immunosensor signal.

## 3. Results and Discussion

### 3.1. Adsorption of Ni Ions to the Organic Films for the Immunosensor

**Figure 3.**
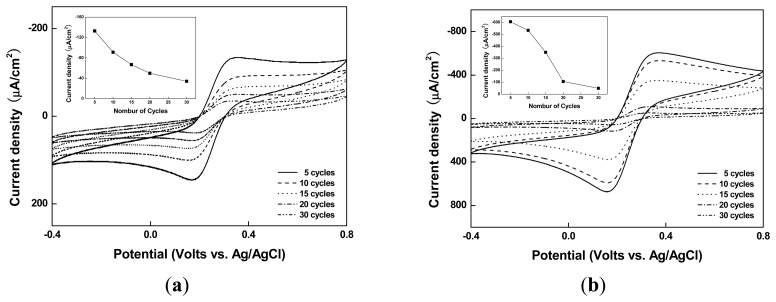
Cyclic voltammograms for (**a**) SPCEs/Organic film and (**b**) SPCEs/Organic film/Ni surface recorded in phosphate buffer solution containing 2 mM K_3_Fe(CN)_6_. Cyclic voltammetry was conducted at a scan rate of 100 mVs^−1^. Inset: Anode currents at 0.375 V *versus* Ag/AgCl as a function of the number of cycles, ranging between five and 30.

**Figure 4.**
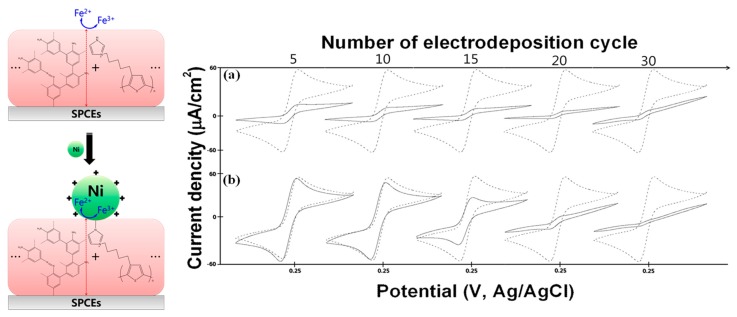
Cyclic voltammograms for: (**a**) SPCEs/Organic film and (**b**) SPCEs/Organic film/Ni surfaces, recorded in phosphate buffer solution containing 2 mM K_3_Fe(CN)_6_. Cyclic voltammetry was conducted at a scan rate of 100 mVs^−1^, and the voltammograms are compared to the bare SPCEs, dashed line.

After forming organic film layers of various thicknesses from p-Phenylenediamine and P3HT-imidazole polymers via electrodeposition cycles and adsorbing Ni ions to each organic film, we added K_3_Fe(CN)_6_ (5 mM) and 1 × PBS (pH 7.4), 20 μL each, and measured signal intensities to test the electron transfer performance of the electrode. The results revealed that as the number of electrodeposition cycles increased, the bare organic film gradually lost signal intensity (Figure 3a), whereas the Ni-immobilized organic film had a steady signal intensity up to 10 cycles, beyond which the signal intensity dropped abruptly (Figures 3b and 4). Covering electrodes with organic films is a superior technology for their protection, which allows elimination of the signals of the interfering substances present in blood or urine. However, the presence of an organic film adversely affects the electron transfer process and thus inhibits the generation of electrochemical signals [35]. While metal nanoparticles are frequently used in order to overcome this drawback, in this study, we developed a method for increasing the electron transfer reaction by binding Ni ions in lieu of metal nanoparticles, taking advantage of the catalytic properties of Ni ions. However, the signal intensity could no longer be maintained after 10 cycles, when the thickness of the organic film layer exceeds 20 Å, whereby Ni ions can no longer contribute to the electron transfer [36]. Given the fact that the signal recovery performance of Ni reaches its limit at a certain thickness of the organic film, we chose 10 cycles as an optimal condition of adsorption of Ni ions to the organic film.

### 3.2. Electrochemical Measurements of the Immunosensor

#### 3.2.1. Immune Reaction between Immobilized Fe-HA and Anti-HA

**Figure 5.**
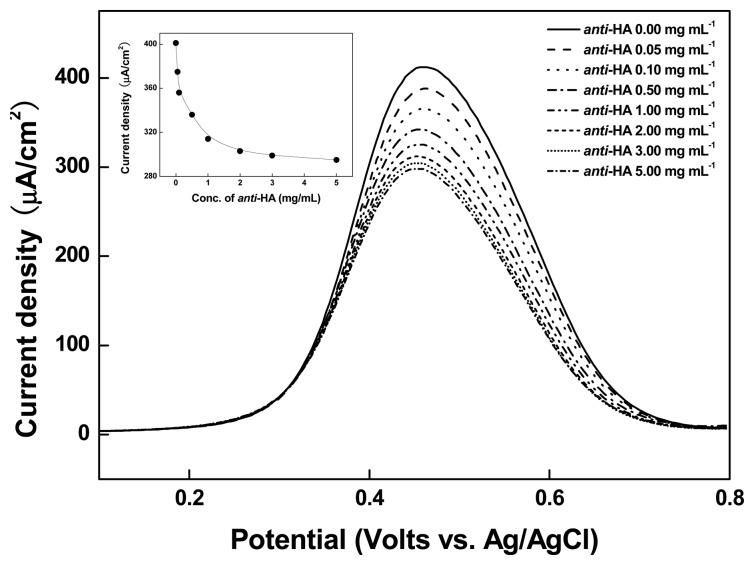
Differential pulse voltammograms of the SPCEs/Organic film/Ni/Fe-HA with variable anti-HA (0 ∼ 5 mg mL^−1^). Inset shows the calibration curve of the cathodic DPV peak current of SPCEs/Organic film/Ni/Fe-HA at 0.45 V *versus* Ag/AgCl as a function of the anti-HA concentrations.

Taking characteristic of the high selectivity and the large molecular mass (∼150,000 Da) of anti-HA, as well as its ability to inhibit the electron transfer of the HA-tagged Fe complex (Fe-HA), we cast anti-HA (40 μL) of different concentrations (0–5 mg/mL) and measured the DPV on SPCEs/Organic film/Ni/Fe-HA. The applied potential ranged from 0.1 to 0.8 V and the scan rate was 50 mV/sec. The curve in the inset graph in Figure 5 shows the dependency of the cathodic peak current fixed at 0.45 V (*versus* Ag/AgCl) on the anti-HA concentration. When the concentration of anti-HA reaches 1 mg/mL, the cathodic peak current sharply decreases and approaches the saturation state of the threshold current in which anti-HA can no longer bind to Fe-HA. The incubation time of anti-HA with the electrode at this point was 20 min [37].

#### 3.2.2. Competition Reaction of Immobilized Fe-HA with Free HA

**Figure 6.**
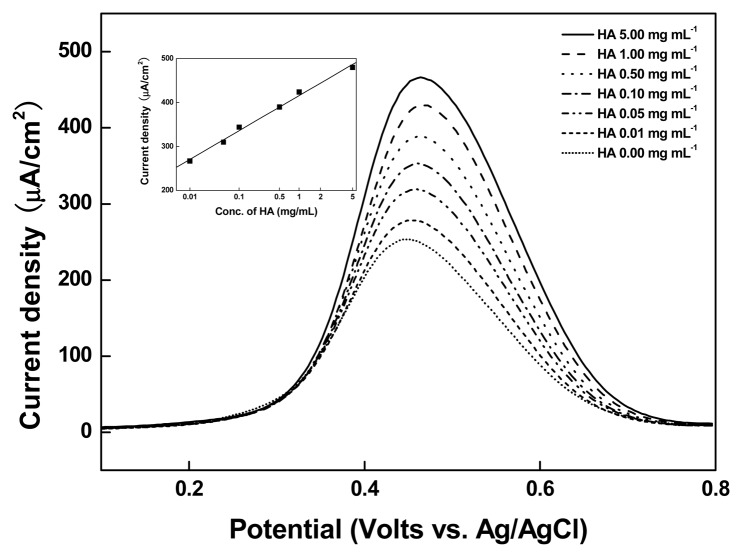
Differential pulse voltammograms of the SPCEs/Organic film/Ni/Fe-HA with variable HA (0 ∼ 5 mg mL^−1^). Inset shows the calibration curve of the anodic DPV peak current of SPCEs/Organic film/Ni/Fe-HA at 0.45 V *versus* Ag/AgCl as a function of the HA concentrations (N = 6, r = 0.9943).

The electrode produced by adsorption of Fe-HA of fixed concentration on SPCEs/Organic film/Ni was casted with 20 μL of HA of various concentrations (0–5 mg/mL) and allowed to react with 20 μL of anti-HA (1 mg/mL) for 30 s and was then checked by DPV. It was verified that, due to the competition reaction between HA and Fe-HA, an increase in the concentration of HA resulted in an increase in its binding with anti-HA. This, in turn, lead to an increase in the number of free Fe-HA thus yielding high current values owing to a smooth electron transfer at the electrode surface. The curve in the inset graph in Figure 6 represents the correlation between the anodic peak current fixed at 0.45 V (*versus* Ag/AgCl) and the concentration of HA. The redox current increased in proportion to the logarithm of the HA concentration of 0.01–5.00 mg mL^−1^, and the limit of detection (LOD) was 5.0 μg mL^−1^ (N = 6, r = 0.9943). Considering that the cutoff concentration of urinary HA is 2.0 mg mL^−1^, the following results indicate the success of electrochemical immunoassay.

#### 3.2.3. Effects of Ascorbic Acid on the Organic Films

**Figure 7.**
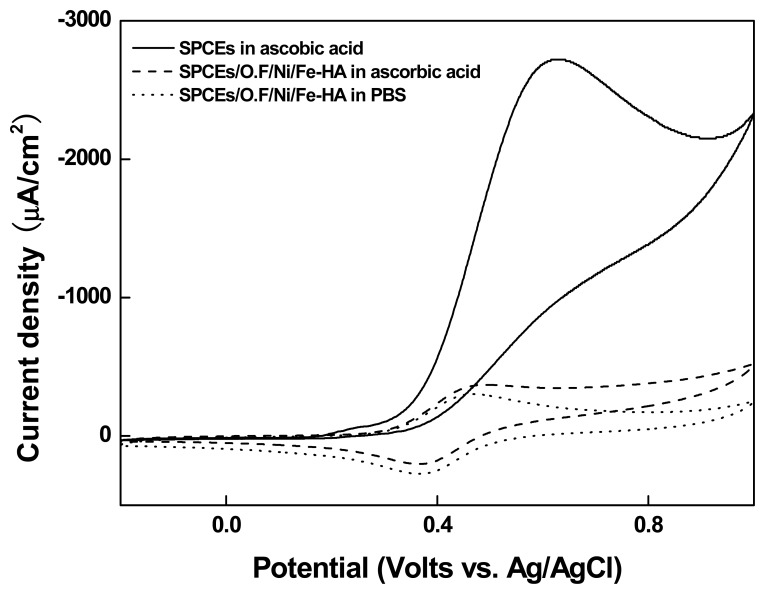
Cyclic voltammogram of the SPCEs/Organic film/Ni/Fe-HA in 2 mM ascorbic acid solution at scan rate of 0.10 V s^−1^.

In order to investigate the potential influence on the electrode of ascorbic acid, which is a common interfering substance in urine, we used cyclic voltammetry to compare the values of the oxidation potential of ascorbic acid in the SPCEs and the same values in SPCEs/Organic film/Ni/Fe-HA (Figure 7). After casting 40 μL of 2 mM ascorbic acid on common SPCEs and SPCEs/Organic film/Ni/Fe-HA, we measured the respective electrical characteristics. In the case of common SPCEs, the oxidation current value of ascorbic acid increased continuously starting from approximately 0.35 V, and the extremely high signal of ascorbic acid interfered with the Fe-HA signal and actually canceled it out. On the contrary, the ascorbic acid signal in the electrode was negligible compared to the signal of Fe-Ha, thus verifying that the interfering substances did not affect the electrode. This may be attributed to the extremely limited electron transfer of ascorbic acid in the presence of the organic film, whereas Ni ions of the organic film present on the surface of the electrode selectively immobilize Fe-HA and display the redox signal of Fe-HA.

## 4. Conclusions

In this study, we formed organic film layers containing imidazole rings on common SPCEs using the electrodeposition method and demonstrated their applicability as electrochemical immunoassay electrodes. Subsequently, exploiting the high affinity between imidazole rings and Ni ions, we immobilized Ni ions on the organic film layers and observed the signal amplification due to the catalytic effects of Ni ions. Additionally, the complex of HA with Fe (an electron transfer mediator) was immobilized on this electrode by means of electrostatic interactions and the latter was applied as an electrochemical immunosensor for toluene detection. By applying the heterogeneous approach to measure the competition between HA and Fe-HA, it was revealed that the electric current values increased in response to an increase in the concentration of HA. Electrodes using organic films can be produced easily and rapidly and applied to a variety of electrochemical immunoassays. They could, therefore, be useful for point-of-care (POC) diagnosis in healthcare.
